# New Data on the Larval Stages of *Leptophallus nigrovenosus* (Digenea, Plagiorchiata)

**DOI:** 10.3390/ani14081154

**Published:** 2024-04-10

**Authors:** Srisupaph Poonlaphdecha, Alexis Ribas, Albert Martínez-Silvestre, Mercedes Villa

**Affiliations:** 1 Parasitology Section, Department of Biology, Healthcare and Environment, Faculty of Pharmacy and Food Science, University of Barcelona, 08028 Barcelona, Spain; spoonlaphdecha@ub.edu (S.P.); mvilla@ub.edu (M.V.); 2 Institut de Recerca de la Biodiversitat (IRBio), Universitat de Barcelona, 08028 Barcelona, Spain; 3 CRARC—Catalonian Reptiles and Amphibians Rescue Center, 08783 Masquefa, Spain

**Keywords:** metacercariae, cercariae, chaetotaxy, amphibian, trematoda, histology, parasite, *Salamandra*, *Planorbarius metidjensis*, urodela

## Abstract

**Simple Summary:**

*Leptophallus nigrovenosus* is a trematode parasite found in snakes that requires feeding on amphibians to acquire the parasite. Firstly, this study investigated, in detail, the sensory receptors on the cercariae of these parasites, shedding light on the adaptations that allow them to find their host species. Secondly, the metacercariae of *Salamandra salamandra* were studied using histology to examine inflammatory responses in the eyes and potential eye damage. This study involved collecting snails (*Planorbius metidjensis*) and observing the emergence of cercariae. Salamander larvae were then exposed to these cercariae, and the larvae were later examined for the presence of metacercariae. Notably, this study is the first to report the presence of *L. nigrovenosus* in the snail *P. metidjensis*, a previously unrecorded host. These findings revealed that members of the genus *Salamandra* can serve as secondary intermediate hosts for *L. nigrovenosus*. Such infections in amphibians can lead to various eye issues, including cloudiness, inflammation, and tissue damage, ultimately affecting their survival. This research highlights the need for further investigation into trematode infections in amphibian eyes, which would provide valuable insights into their prevalence, transmission dynamics, and impact on host populations.

**Abstract:**

(1) Background: *Leptophallus nigrovenosus*, an esophageal parasite that primarily affects water snakes of the genus *Natrix*, has a known life cycle that involves snail and amphibian hosts. However, the biological aspects, chaetotaxic patterns, and pathogeny of this parasite in its hosts have not been fully elucidated. (2) Methods: Snails (*Planorbarius metidjensis*) were collected in Spain and examined for cercaria emergence. The larvae were used to experimentally infect *Salamandra salamandra*, and metacercariae were isolated. Their chaetotaxy was studied using microscopy and scanning electron microscopy. The eye histology was also examined. (3) Results: The cercariae displayed distinctive morphological characteristics. The results of this study revealed three types of ciliated sensory papillae on the cercarial teguments, suggesting an adaptation for host detection and orientation. The metacercariae isolated from subcutaneous tissues showed oval bodies covered in spines. The chaetotaxy patterns matched those of Leptophallinae species. This is the first report of the presence of *L. nigrovenosus* in the snail *P. metidjensis*. Additionally, this study detected metacercariae in the eyes of *S. salamandra*, emphasizing the need for further research on trematode infections in amphibian eyes. (4) Conclusions: Members of the genus *Salamandra* can serve as secondary intermediate hosts for *L. nigrovenosus*, and the presence of metacercariae in amphibian eyes may have implications for the survival and habitat management of these amphibians. Understanding this parasite’s prevalence, transmission dynamics, and impacts on host populations is crucial for conservation strategies.

## 1. Introduction

**Figure 1:**
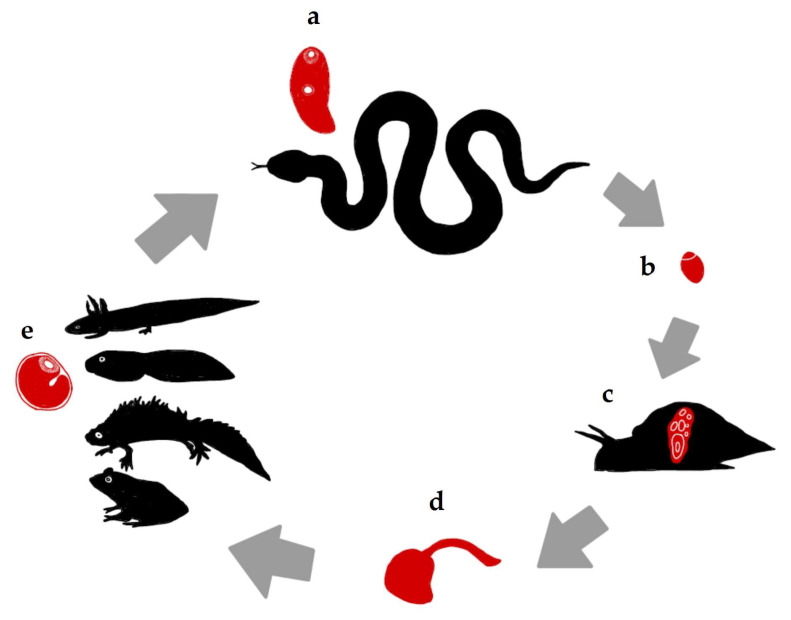
The life cycle of *Leptophallus nigrovenosus* according to a literature compilation and our findings. (**a**) Definitive host: Ophidians are the main definitive hosts of the adult trematodes; they acquire the parasite by feeding on amphibians. (**b**) Eggs expelled in the feces of reptiles reach a body of water. (**c**) The first intermediate host: After the eggs are ingested, the miracidium leaves the egg shell in the snail intestine, develops into a sporocyst, and starts to produce cercariae (asexual multiplicative reproduction). (**d**) The cercariae are shed from the snail and actively search for a second intermediate host (amphibians). (**e**) The first intermediate host: Anurans and urodeles (larvae and adults) in contact with water harbor the metacercariae as a result of cercariae penetration in different parts of their bodies.

The adult digenean *Leptophallus nigrovenosus* (Bellingham, 1844) is an esophageal parasite of ophidians in Europe and northern Africa and is mainly found in water snakes of the genus *Natrix* (Laurenti, 1768) [1,2]. The infective stage for snakes (metacercaria) is found in amphibians (adults and tadpoles) that comprise some of the main prey species in the diet of *Natrix* snakes [3]. This trematode has been recorded across Europe in different representatives of the genus *Natrix*: the grass snake *Natrix natrix* s.l. (L., 1758) in Georgia [4], Hungary [5,6], Italy [7], Poland [8,9], Romania [10], Russia [11,12], Spain [13,14], and Ukraine [15]; the dice snake *Natrix tessellata* (Laurenti, 1768) in Hungary [5] and Russia [16]; and the viperine water snake *Natrix maura* (L., 1758) in Spain [13]. The life cycle of *L. nigrovenosus* (Figure 1) is already known, as described in [17], although this author and the author of [1] acknowledged that the cercariae, metacercariae, and adults were described earlier by the Italian veterinarian Giovanni Battista Ercolani in his 1881 and 1882 works.

In France, the author of [17] described subcutaneous metacercariae, found in the tadpoles of *Rana* sp., and adult trematodes, obtained through the experimental infection of grass snakes (*N. natrix*). It was observed that the penetration of cercariae and the posterior development of metacercariae into tadpoles occur in different parts of the body [17,18]. The first intermediate hosts found by this author in France were the great pond snail (*Lymnaea stagnalis* (L., 1758)) and *Ampullaceana ampla* (Hartmann, 1841) (named *Radix ampla* in the original study) (Lymnaeidae). Later, the author of [19] detected *L. nigrovenosus* cercariae in the lymnaeid *Ampullaceana balthica* (L., 1758) (named *Lymnaea limosa* in the original study) in the French oriental Pyrenees. In Poland, *L. nigrovenosus* was detected in *A. balthica* (named *Radix ovata* in the original study) (Lymnaeidae) as the first intermediate host, and in the tadpoles of the moor frog *Rana arvalis* (Nilsoon, 1842) (named *Rana terrestris* in the original study) and the edible frog *Pelophylax* kl *esculentus* (L., 1758) (named *R. esculenta* in the original study) as the second intermediate hosts; *L. nigrovenosus* completed its life cycle in these hosts [20,21]. This author also reported the experimental life cycle of other representatives of this subfamily (*Metaleptophallus gracillimus* (Lühe, 1909)), starting with cercariae that naturally parasitize the planorbid snail *Planorbarius corneus* (L., 1758) and then infect tadpoles of *R. arvalis* (named *R. terrestris* in the original study) and the common frog (*R. temporaria* L., 1758) in order to obtain mature metacercariae; then, the adults are reared in grass snakes (*N. natrix*) [20]. Reports also exist on definitive hosts other than aquatic snakes, including the common European adder *Vipera berus* (L., 1758) in Belarus [22], Hungary [5], Russia [11,12,23], and Ukraine [18]; despite this snake’s terrestrial habitats, it is a generalist predator that feeds on amphibians [24]. In addition, adult digeneans have been reported in the lizards *Lacerta viridis* (Laurenti, 1768) in Bulgaria [25] and *Lacerta agilis* (L., 1758) in Russia [12]. Although they consume mainly insects [26], they may opportunistically feed on amphibians in some situations. Additionally, the following second intermediate hosts (characterized by the presence of metacercariae in an amphibian) have been reported: the European fire-bellied toad (*Bombina bombina* (L., 1758)), the yellow-bellied toad (*Bombina variegata* (L., 1758)), the common toad (*Bufo bufo* (L., 1758)), *R. arvalis, R. temporaria*, and the smooth newt *Lissotriton vulgaris* (L., 1758) in the former Czechoslovakia [27]; the northern crested newt (*Tritus cristatus* (Laurenti, 1768)) in Russia [11]; and the alpine newt (*Ichthyosaura alpestris* (Laurenti, 1768)) in Hungary [6]. Additional records have identified *Stagnicola palustris* (O. F. Müller, 1774) [28] in Poland and *Radix peregra* (O.F. Müller, 1774) in the Czech Republic [29] as first intermediate hosts. Studies on the biology of other leptophallids of different genera (*Macrodera* (Loos, 1899) and *Paralepoderma* (Dollfus, 1950)) have shown that adult parasites are found in snakes and different species of planorbiid snails, and that tadpoles act as intermediate hosts for these parasites [30,31,32,33].

The chaetotaxic patterns of all of the above-mentioned species of leptophallids have been studied or reviewed [19,21,34]. In order to identify *Leptophallus* species, complementary information is needed, including a description of the entire life cycle, the chaetotaxic pattern of the cercariae, and the morphology and habitat of the adults, and molecular tools should also be used [35].

In Spain, within our study area, adult *L. nigrovenosus* has been recorded in *N. natrix* and *N. maura* [13]; however, despite the fact that the terrestrial snakes in this area were surveyed for parasites, *L. nigrovenosus* trematode adults were not found [36]. Regarding the intermediate hosts, the metacercariae of this species have been found in the European tree frog (*Hyla arborea* (L., 1758)) [13]; Perez’s frog (*Pelophylax perezi* (López-Seoane, 1885)) [13,37]; the Iberian midwife toad (*Alytes cisternasii* (Boscá, 1879)) [13]; and the Pyrenean brook salamander (*Calotriton asper* (Dugès, 1852)) [38]. However, information on the first intermediate host is lacking.

## 2. Materials and Methods

### 2.1. Sample Collection and Cercaria and Metacercaria Isolation

Snails (*Planorbarius metidjensis*) were collected in Grazalema, in the south of Spain, and transported to the parasitology laboratory at the University of Barcelona. A total of 116 snails were placed individually in cell culture plates containing 5 mL of filtered biotope water for the detection of cercaria emergence. The experimental conditions were as follows: temperature, 15 °C; photoperiod, 12 h/12 h light/darkness; and relative humidity, 80%. The plates were examined daily for 23 consecutive days (three times a day) under a stereomicroscope to detect spontaneous cercaria emissions. For the infestation of salamander larvae, a total of 80 recently emerged cercariae were isolated from the positive well of the culture plate with a Pasteur pipette and transferred to a Petri dish with 1 cm of water. Three *Salamandra salamandra* larvae, reared in the laboratory from eggs and fed with frozen chironomid larvae, were added to the container and exposed for ten hours at 22 °C. No cercariae were observed in the container after the 10 h. The salamanders were anesthetized, subsequently killed using MS222 (ethyl-4-aminobenzoate), and necropsied to obtain metacercariae at 25 days (1 individual, 37 isolated metacercariae) or 60 days (2 individuals, 30 isolated metacercariae) post-infection. The collection of metacercariae was achieved by crumbling the tissues of the salamander larvae using tweezers and a scalpel, placing the crumbled tissues into a Petri dish with saline solution, and observing them under a stereomicroscope. The recovered metacercariae were fixed in Bouin’s fluid between a slide and a cover glass (without pressure), subsequently colored with carmine, and mounted in Canada balsam. In addition, the eyes were fixed in Bouin’s fluid for the histological study. Both the fixed and living materials were studied microscopically using a Leitz Laborlux K microscope, and figures were made with the aid of a camera lucida. Microphotographs were obtained using Wild Leitz MPS 46 Photoautomat equipment, Ernst Leitz GmbH, Wetzlar, Germany.

### 2.2. Cercarial Sensory Receptors

**Figure 2:**
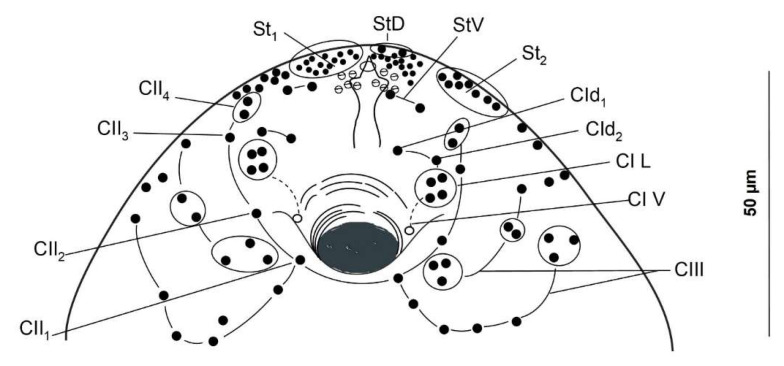
The distribution of the integumentary ventral sucker papillae on a cercaria of *Leptophallus nigrovenosus*.

**Figure 3:**
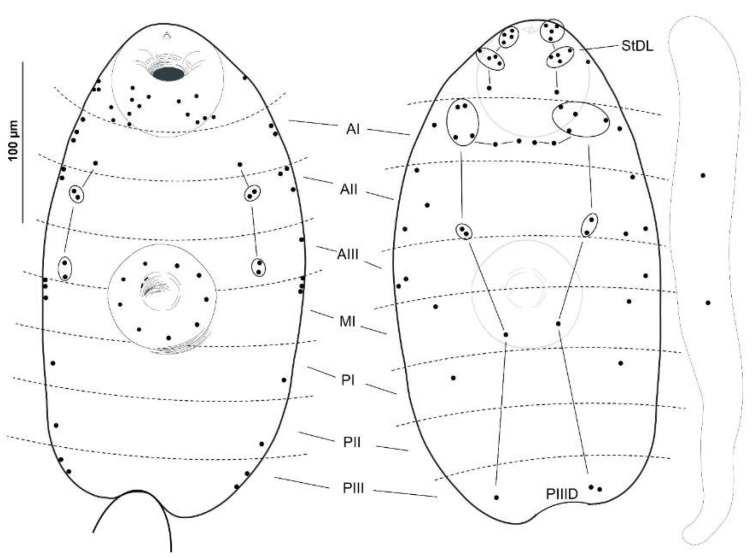
The distribution of the integumentary papillae on a cercaria of *Leptophallus nigrovenosus*: ventral surface, dorsal surface, and tail, from left to right.

**Figure 4:**
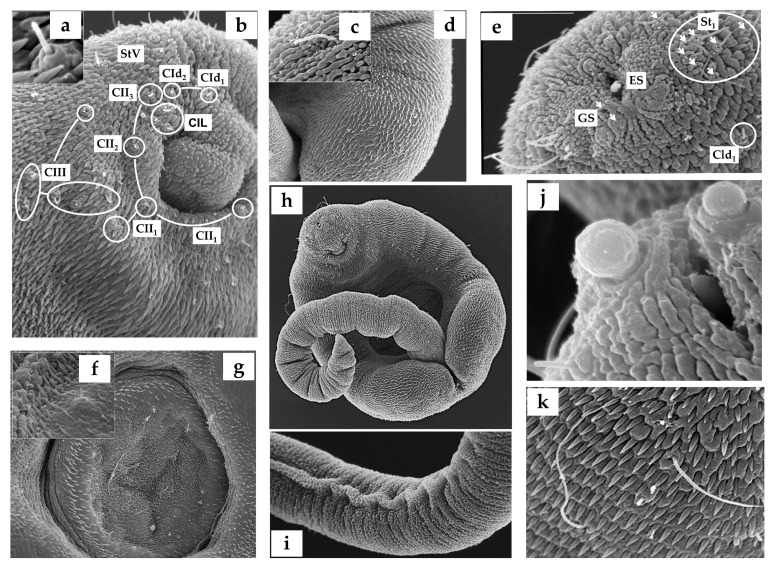
The patterns of argentophilic papillae on the scanning electron micrographs of *L. nigrovenosus* cercariae: (**a**) the morphology of the integumental cephalic ring papillae; (**b**) the anterior ventral surface and (**c**,**d**) posterior lateral view of a cercaria, showing medium sensilla; (**e**) the stylet’s integumentary surface; (**f**,**g**) the details of small dome sensilla on a ventral sucker; (**h**) the ventral topographic surface of a cercaria; (**i**) the tail region; (**j**) the secretory penetration glands; and (**k**) the dorsal anterior integumental long sensilla.

The distribution patterns of cercaria sensilla were studied using both chaetotaxy maps (Figure 2 and Figure 3) and scanning electron microscopy in 20 specimens (Figure 4). For the argentophilic impregnation of papillae, the cercarial chaetotaxy was studied in 20 specimens. The methodology employed was based on that described in [39]; thus, for the argentophilic impregnation of papillae, the cercariae were rinsed in distilled water, stained with cold 3% silver nitrate, exposed to natural light, washed in distilled water, and mounted in Faure’s chloral gum medium. The nomenclature of the papillae was assigned according to [19,21].

To examine the cercariae using scanning microscopy, the specimens were fixed in 2.5% glutaraldehyde in Sorensen’s buffer (pH of 7.4), postfixed in 1% osmium tetroxide in Sorensen’s buffer (pH of 7.4), dehydrated in acetone, coated with gold, and examined using a Hitachi S-2, 300 scanning electron microscope (Hitachi, Ltd. Chiyoda, Japan) operating at 15 kV.

Both the fixed and living materials were studied microscopically using a Leitz Laborlux K microscope (Ernst Leitz GmbH, Wetzlar, Germany), and figures were made with the aid of a camera lucida. Microphotographs were obtained using Wild Leitz MPS 46 Photoautomat equipment.

### 2.3. Histology

**Figure 5:**
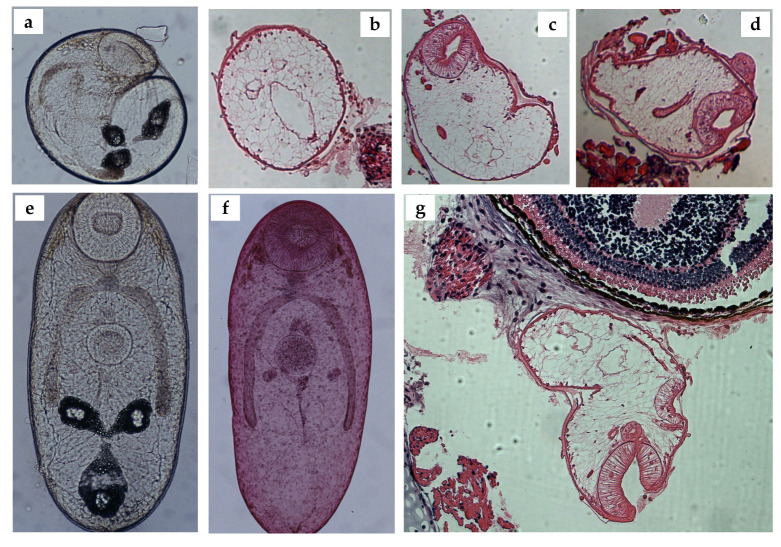
(**a**) An encysted mature metacercaria from a salamander eye (direct observation in saline solution), at 25 days post-infection; (**b**–**d**) metacercariae observed using histology, at 25 days post-infection; (**e**) an excysted metacercaria at 25 days post-infection (direct observation in saline solution); (**f**) an excysted metacercaria at 25 days post-infection, stained with Semichon’s carmine; and (**g**) a metacercaria at 25 days post-infection, located in the retrobulbar choroidal space, close to the sclerotia and surrounded by fibrous tissue and a low inflammatory reaction.

Eye histology was performed according to the standard automated system; the eyes were fixed in 4% buffered formaldehyde, embedded in a TEC2900T-1 paraffin system, and cut in 5 μm thick sections using a KD-3358 semi-automatic microtome. The sections were deparaffinized, rehydrated, and stained (Masson’s trichrome stain) in a KD-RS5 automatic tissue processor (Figure 5).

## 3. Results

### 3.1. Cercariae

Of the 116 examined snails, two shed cercariae in small numbers, reaching up to 80 cercariae per day from one infected gastropod. The usual range was between 1 and 31 cercariae per day. These larvae were reluctant to swim; after emerging, they fell to the bottom of the plate, remaining in a resting position. Then, they swam vertically towards the surface with the aid of a vigorous movement of the tail, which twisted perpendicularly to the body axis, and the body was curled ventrally so that the oral sucker touched the base of the tail. The cercariae were large and thick, and their parenchyma contained numerous granules, particularly in the central zone, where the cystogenous glands were located. Small tegumental spines covered the entire forebody and were more dispersed on the dorsal side. The tail was laterally flattened with narrow dorsoventral finfolds. The suckers, both oral and ventral, were muscular and nearly of the same size. Four pairs of penetration glands were displayed laterally on the body at the anterior level (in front) of the ventral sucker. The stylet was simple and slender, with a short point and without transversal thickening. The excretory vesicle was thick-walled and Y-shaped, with arms of equal length. The digestive system was well developed, with the intestinal caeca reaching the excretory vesicle. We aimed to use a combination of silver nitrate impregnation and scanning electron microscopy (SEM) transmission to map the network of nervous endings on the cercarial tegument (Figure 2 and Figure 4). Likewise, this distribution of sensors on the surface allowed us to contrast it with other already-known chaetotaxy maps (see Table 1).

The present study revealed the presence of three types of ciliated sensory papillae on the cercarial tegument, disposed in bilaterally symmetrical lines and distinguished by the length of the cilium-like structure and their surface position. These included a long cilial receptor (12-micron length), disposed on the anterior dorsal side, and two types surrounded by a tegumentary collar, with a (2) moderately long (5-micron length) or (3) short (2.5-micron length) cilium. These latter two types were found mainly in the acetabulum and ventral sucker regions.

### 3.2. Metacercariae

The isolated metacercariae from the subcutaneous tissue at 25 days post-infection were completely developed inside thin, oval-shaped cysts (Figure 4a); no differences were observed between these metacercariae and those obtained at 60 days post-infection. The bodies of the excysted metacercariae were oval-shaped, and the whole surface of the tegument was covered with small spines. The oral sucker was subterminal and larger than the ventral sucker. Around the oral sucker, several glands were observed. The digestive system was well developed: the prepharynx and esophagus were short; the pharynx was muscular and well developed; and the narrow intestinal caeca reached the excretory vesicle. The Y-shaped excretory vesicle was prominent and had arms of equal length. The cell complex that formed the primordia of the ootype and the ovary was located between the ventral sucker and the arms (anus) of the excretory vesicle. The primordium of the testis was situated laterally, at one level, on the dorsal side of the body. The cells forming the uterus led to the ootype, descending from the end of the caeca and ascending to the edge of the ventral sucker, where they connected to the primordia of the genital atrium and cirrus pouch. The primordia of the vitellaria were formed by small preacetabular glands, which were clearly visible. A histological analysis of the eyes revealed the presence of metacercariae (Figure 5b–d,g).

## 4. Discussion

In this study, the features of our cercariae matched those displayed by the cercariae of *L. nigrovenosus* [19,34]; the chaetotaxy also fit with that of other representatives of Leptophallinae for which data are available (*Metaleptophallus gracillimus*, *Macrodera longicollis* (Abildgaard in Müller, 1789), *Paralepoderma brumpti* (Buttner, 1951), and *Paralepoderma progeneticum* (Buttner, 1951); details shown in Table I). Following the nomenclature proposed in [21], a chaetotaxical comparison between our cercariae and the ones mentioned above showed similarities, as noted by [34] among their cercariae. These similarities included the following: (a) the general distribution of papillae in the cephalic rings (C); (b) the AID composed of a transverse row of four median papillae, flanked on both sides by four sensilla; (c) dorsal (one PI and one PIII) and ventral (one AI, two AII, two AIII, one PI, one PII, and one PIII) body sensors, although the authors noted that sometimes PII and PIII were lacking; and (d) ventral sucker sensors (nine constant SI and a variable number of SII; zero to six papillae). These common features were also observable in our individuals. As previous authors [34] have affirmed, *L. nigrovenosus* and *M. gracillimus* are closely related, but differ in the following features: (a) the sensilla of the cephalic rings (one CII1, one CIV-V4, and one CIV-V5 in L.n. vs. two to three CII1, two CIV-V4, and two CIV-V5 in L.g.), and (b) the papillae at the acetabular level (nine SI and zero SII in *L. nigrovenosus* vs. nine SI and five to six SII in *M. gracillimus*). In this sense, our cercariae matched exactly with the features displayed by *L. nigrovenosus*, thus being more closely related to this species than to *M. gracillimus*. Slight differences were observed among the cercariae from the three studied localities (France, Poland, and Spain), but the influence of the method of description used must be taken into account. The chaetotaxy of the cercariae from the French locality was studied in [19] and showed a similar pattern to that of the present study, with the main differences as follows: (a) cephalic level: 14 papillae in this study (2 arcs of 7) instead of the 13 in [19] (arcs of 6 and 7) and a superior number of St1 papillae in this study (only 12 in [19]), and (b) body level: no ventral sensors in PI in the Spanish cercaria, compared to one PIV in [19]. When examining the drawings made by the different above-mentioned authors, a controversial criterion is evident in the ventral versus lateral position of the sensilla of the posterior body levels P. In the Polish samples [34], one PIV, one PIIV, and one PIIIV were displayed; in the cercariae examined in [19], only one PIV was observed, along with lateral PII and PIII papillae; finally, our cercariae did not clearly display any ventral papillae, but did display latero-ventral papillae at these levels.

According to the authors of [34], the cephalic regions of *L. nigrovenosus* bear 56–62 sensilla, of which 26–28 are circumoral (7 CI, 7 CII, 6–7 CIII, and 5 CIV) and 30–33 are stylet sensilla, whereas the *L. nigrovenosus* studied in [19] presented 58–59 sensilla, with 25 being circumoral (7 CI, 5 CII, 7 CIII, and 5 CIV) and 33–37 being stylet sensilla. The present study found more total cephalic papillae, with 62–63 sensilla, including 26 circumoral (7 CI, 5 CII, 8 CIII, and 6 CIV) and 37–38 stylet sensilla. The characters that seem to be common to *L. nigrovenosus*, *L*. (=*M*.) *gracillimus*, and our cercariae are the number and distribution of cephalic papillae of CI (seven in all three) and the conflictive papillae ventral and lateral to the P levels. The cercariae examined in the present study could be characterized based on the disposition of the cephalic rings, CIII and CIV, and the more para-dorsal disposition of one of the two lateral papillae of PI.

The presence of sensorial receptors in cercariae is an adaptation that increases the chances of the cercariae encountering their host spectra [40]. The sensory receptors with moderately long and short unciliated sensilla located on the anterior body, including the ventral sucker, are likely intended to locate and recognize the most suitable sites for host penetration. Furthermore, the sensory papillae with shorter cilia may be more sensitive to different pressures, suggesting a tango-/mechanoreceptive function to perceive the host once the cercariae touch the host [41,42]. Some receptor types were site-specific, such as the long, unciliated receptors located in the anterior dorsal region of the cercarial body, which could serve to orient the cercariae in the water column by alternating between swimming and resting periods [43] or could also serve as drag anchors during resting periods [44].

To our knowledge, there have been no previous reports on the presence of *L. nigrovenosus* in the genus *Salamandra*. From an ecological point of view, *Salamandra* species appear to be well adapted to this parasite’s life cycle, as salamander larvae share the same habitat as and are eaten by *Natrix* species [3]. In addition, this is the first report of the snail *P. metidjensis* as a host for this parasite, as this genus was absent from the review in [45].

The observed metacercariae caused a fibrinous reaction in the surrounding host tissues, which is a characteristic inflammatory response of amphibians to an infection, parasite, or foreign body [46]. Although there was no internal affectation of the ocular structure in this case (neither the anterior nor the posterior chamber was affected), nor an abundance of inflammatory cells, this proliferation of fibrous tissue would hinder the correct functioning of the affected eye (through abnormal movement, pain, or malposition), causing the symptoms we described before and making it more difficult for the affected animal to survive in its habitat. The pathology and clinical manifestations of trematode infections in the eyes of amphibians can lead to a range of pathological changes, including corneal opacity, inflammation, peripheral abscesses affecting glandules or adipose tissue, and even damage to internal ocular tissues. As a result, infected amphibians may exhibit symptoms such as cloudy or swollen eyes, abnormal behavior, and reduced visual acuity. Severe infections can ultimately lead to permanent eye damage and impaired survival in the wild [47,48]. This is the first report on the presence of metacercariae in the retrobulbar tissues of salamanders. Furthermore, the presence of this parasite could hinder this amphibian’s ability to evade its predators. In this way, it would be easier for the parasite to complete its life cycle in predatory snakes.

The fire salamander (*S. salamandra* (L., 1758)) faces several conservation threats; over the last few decades in Spain, it has undergone population regressions in several regions [49]. These conservation threats include land use changes, where this amphibian disappears in intensively deforested areas, plantations, and monocultures [50]. Under climate scenarios related to climate change for the 21st century, models have projected contractions in the current potential distribution of this species by 49% to 53% in 2041–2070. The level of coincidence between the observed and potential distributions is projected to decrease to a range of 50% to 53% in 2041–2070 [51]. Other anthroponotic factors, such as roadkill rates, affect the populations of this amphibian [52].

Furthermore, emerging pathogens that threaten this species have been detected in recent years in the Spanish Caudata. The fire salamander is affected by several parasitic diseases, including emerging diseases. A wide spectrum has been found that includes bacteria, viruses, fungi, and helminths. Cultures and PCRs performed in Spain for the detection of the bacterium *Chlamydia* (Jones et al., 1945) (Chlamydiaceae) have been negative so far for both wild and captive populations [53]. However, Ranavirus (Iridoviridae) is more frequent, according to the surveillance conducted on Spanish salamanders and other amphibians [54]. The fungi *Batrachochytrium dendrobatidis* (Longcore, Pessier, and D.K. Nichols (1999)) and *Batrachochytrium salamandrivorans* (Martel A., Blooi M., Bossuyt F., Pasmans F. (2013)) (Batrachochytriaceae), which produce chytridiomycosis, have been detected in Spain [55], and experimental studies have shown that these amphibians are at risk [56]. Since 2018, an outbreak of the deadly fungus *B. salamandrivorans* has been detected in Barcelona. Carriers and dead specimens were detected in both newts and salamanders, and several measures have been applied since then to prevent the spread of this pathogen [57]. In Spain, several helminths have been detected in this urodele, including the acantocephalan *Acanthocephalus anthuris* (Dujardin, 1845) (Echinorhynchidae) [13]; the digenean trematodes *Brachycoelium salamandrae* (Fröelich, 1789) (Brachycoeliidae) [58] and *Haematoloechus carbonelli* (Lluch, Navarro, and Pérez–Soler, 1991) (Haematoloechinae) [59]; and the nematodes *Dorylaimus parasiticus* (Navarro, Guerrero, Pérez-Mellado, and Lluch, 1995) (Dorylaimidae) [60], *Rhabdias bufonis* (Schrank, 1788) (Rhabdiasidae) [11], and *Oxysomatium brevicaudatum* (Schneider 1866) (Cosmocercidae) [58]. Recently, a novel disease with an unknown etiological agent (probably a protist-like organism) has been described in salamanders from Italy [61].

The effects of the nematode *Rhabdias* (Stiles and Hassall, 1905) on its host appear to be primarily sublethal; however, a dose-dependent reduction in growth and an overall impaired locomotor performance still represent a significant reduction in host fitness [62].

Several factors have been explored with regard to their interactions with the parasites of amphibians. Pollution by microplastics has been proven to increase the susceptibility of *S. salamadra* larvae to the chytrid fungus *B. dendrobatidis* [63].

In the case of trematodes, agrochemicals (atrazine and phosphate) have been proven to increase the exposure and susceptibility of amphibians to larval trematodes [64]; exposure to the pollutant NaCl can also alter the relative susceptibility of host species to trematodes [65]. The pathogenic and health-status effects of trematodes on amphibians have been demonstrated in several species. The adult lung fluke (*Haematoloechus* spp.), commonly found in adult frogs, is now known to cause substantial tissue damage in its host [66]. The genus *Ribeiroia* (Travassos, 1939) (Psilostomatidae) is a teratogenic trematode; the cercariae of this species encyst in larval amphibians, producing limb malformations, including multiple extra limbs or partially missing limbs [67]. In addition, laboratory experiments on *Echinostoma* (Rudolphi, 1809) (Echinostomatidae) have shown that this parasite can cause extensive mortality in tadpoles [68]. The P-syndrome hotspot found in tadpoles and metamorphs of water frogs and toads in Europe is due to parasitization by metacercariae of the trematode *Strigea robusta* (Szidat, 1928) (Strigeidae), which results in various deformities of the hindlimbs and forelimbs [69]. L. nigrovenosus metacercariae has been reported with a high prevalence (22.2%) in *Pelophylax saharicus* (Boulenger, 1913) in Morocco [37]. It should be noted that this anuran has been detected as an introduced population in the south of France [70], so future research should address the possible contribution of this amphibian to the maintenance of the life cycle in Europe. In conclusion, the study of trematode life cycles and their host pathologies can contribute to the conservation of amphibians.

Future research on trematode infections in amphibian eyes would provide insights into these parasites’ prevalence, transmission dynamics, and impact on host populations. In light of the documented pathologies caused by trematodes and their role as bioindicators, we strongly emphasize the need for further comprehensive research on this understudied group, as previously advocated by the authors of [71].

## 5. Conclusions

The genus *Salamandra* can serve as a secondary intermediate host for *L. nigrovenosus* following the natural penetration of cercariae, resulting in the potential development of pathology.

Understanding aspects of this parasite’s prevalence, transmission dynamics, and impact on host populations is crucial for implementing effective conservation strategies, including the monitoring and management of amphibian populations in affected habitats.

## Figures and Tables

**Table 1:** A comparison of the chaetotaxy of the cercariae in our study with those of the five studied Polish species according to the nomenclature in [21]. *L.n*: *L. nigrovenosus*; *M.g.: M. gracillimus*; *M.l.: M. longicollis*; *P.b.: P. brumpti*; and *P.p.: P. progeneticum*. Bold numbers: the papillae from the stylet levels according to Richard’s nomenclature. * The ventral papillae were displaced laterally (see text). ** According to the nomenclature in [19], Mil I dorsal region.

	Present Study	Grabda-Kazubska and Bayssade-Dufour (1999) Poland				
	*L. n.*	*L. n.*	*M. g*	*M. l.*	*P. b.*	*P. p.*
Cephalic						
CI	11; 02; 43; 14; 15	11; 02; 43; 14; 15	11; 02; 43; 14; 15	1–21; 02; 4(3)3; 14; 15	1–21; 0–12; 43; 14; 15	1–21; 12; 43; 14; 15
CII	11; 12; 13; 24; 20–225	11; 32; 13; 24; 18–225	2–31; 22; 23; 24; 17–215	11; 2(3)2; 33; 24; 18–255	21; 22; 23; 24; 16–225	21; 2(3)2; 23; 24; 16–245
CIII	21; 32; 33; 6–84; 35	21; 2–32; 23; 5–84; 35	21; 22; 23; 3–54; 3(4)5	21; 12; 23; 3–54; 45	21; 2(3)2; 2–33; 5–74; 3–45	2(1)1; 2(3)2; 2–43; 4–94; 35
CIV	21; 32; 13–4; 45	21; 22; 13–4; 45	21; 22; 2(0)3; 24; 3–45	21; 22; 23; 4–74; 45	2–31; 1–22; 1–33; 2–44; 45	1–31; 1–22; 2(3)3; 3–64; 45
CIV-V	14; 15	14; 15	24; 25	14; 05	1(2)4; 1(2)5	1(2)4; 1(2)5
Body—Dorsal						
AID	2; 4	2; 4	2; 4	2; 4(3)	2; 4	2; 4(3)
AIID	2	2	1	1	2	2
AIIID	-	-	1	1	-	-
PID	1 (=MID) **	1	1	1	1	1
PIID	-	-	-	-	-	-
PIIID	1	1	1	1	1	1
Body—Ventral						
AIV	1	1	1	1	1	1
AIIV	2	2	2	2	2	2
AIIIV	2	2	2	1(2)	2	2
PIV	- *	1	1	1	1	1
PIIV	- *	1	1	-	(1)	(1)
PIIIV	- *	1	(1)	-	-	-
Body—Lateral						
AI to PIII	17–18	16–21	18–24	24–27	18–23	18–26
Acetabulum						
SI	9	9	9	9	9	9–11
SII	-	-	5–6	0–4	0–3	0–6
Caudal						
UD	2	2	2	2	2	2

## Data Availability

The data are contained within the article.
